# Verbal and visuospatial executive functions in healthy elderly: The impact of education and frequency of reading and writing

**DOI:** 10.1590/S1980-57642014DN82000011

**Published:** 2014

**Authors:** Laura Damiani Branco, Charles Cotrena, Natalie Pereira, Renata Kochhann, Rochele Paz Fonseca

**Affiliations:** 1 Graduate Psychology Program - Department of Psychology - Pontifical Catholic University of Rio Grande do Sul - PUCRS. Porto Alegre, RS – Brazil.

**Keywords:** aging, education, reading, writing, executive function

## Abstract

**Objective:**

To assess the predictive role of education and frequency of reading and writing habits (FRWH) on the cognitive flexibility, inhibition and planning abilities of healthy elderly individuals.

**Methods:**

Fifty-seven healthy adults aged between 60 and 75 years with 2 to 23 years of formal education were assessed as to the frequency with which they read and wrote different types of text, as well as their number of years of formal education. Executive functions were evaluated using the Hayling Test and the Modified Wisconsin Card Sorting Test (MWCST).

**Results:**

Weak to moderate positive correlations were found between education, FRWH and the number of categories completed in the MWCST, while negative correlations were identified between these variables and the number of perseverative and non-perseverative errors on the task. Only the FRWH was significantly correlated with the number of failures to maintain set. Speed and accuracy on the Hayling Test were only correlated with participant education. Both education and FRWH significantly predicted performance on the MWCST, and the combination of these two variables had a greater predictive impact on performance on this task than either of the two variables alone. Variability in scores on the Hayling Test was best accounted for by participant education.

**Conclusion:**

In this sample of elderly subjects, cognitive flexibility was sufficiently preserved to allow for adequate performance on verbal tasks, but may have benefitted from the additional stimulation provided by regular reading and writing habits and by formal education in the performance of more complex non-verbal tasks.

## Abstract (pt)

**Objetivo:**

Avaliar o papel preditivo da educação e da frequência de hábitos de leitura e de escrita (FHLE) na flexibilidade cognitiva e planejamento de idosos saudáveis.

**Métodos:**

Cinquenta e sete adultos saudáveis com idade entre 60 e 75 anos e de 2 a 23 anos de escolaridade foram avaliados quanto à frequência com que liam e escreviam diferentes tipos de texto, assim como seu número de anos de educação formal. Ainda, suas funções executivas foram examinadas pelos Testes Hayling e Wisconsin de Classificação de Cartas Modificado (MWCST).

**Resultados:**

Correlações positivas de fracas a moderadas foram encontradas entre a educação, a FHLE e o número de categorias completadas no MWCST. Ainda, estas variáveis correlacionaram-se negativamente com o número de erros perseverativos e não perseverativos na tarefa. Somente a FRWH correlacionou-se com o número de rupturas no MWCST. O tempo e acurácia no Teste Hayling correlacionaram-se apenas com a educação de participantes. Tanto a educação quanto a FHLE foram preditores significativos do desempenho no MWCST, e a combinação destas duas variáveis teve maior impacto no desempenho da tarefa do que qualquer uma delas isoladamente. A variabilidade no desempenho no teste Hayling foi melhor explicada pela educação dos idosos.

**Conclusão:**

Nesta amostra de indivíduos idosos, a flexibilidade cognitiva esteve suficientemente preservada para permitir um desempenho adequado em tarefas verbais. No entanto, pode ter se beneficiado do estímulo adicional fornecido por hábitos de leitura e escrita regulares e por maior quantidade de anos de estudo formal em demandas não-verbais mais complexas.

## INTRODUCTION

The role of individual and sociocultural factors in cognition has been a major focus of study in neuropsychology,^1,2^ as has the influence of aging on cognition.^3-5^ Most neuropsychological investigations of the impact of sociodemographic factors on cognitive ability have focused on the role of education,^6^ socioeconomic status,^7^ sex/gender,^8^ and, in more recent studies, of the frequency of reading and writing in cognitive performance.^9,10^

Education, usually defined as the number of years of formal study completed, has proved to be an important determinant of neuropsychological performance.^11-15^ Higher educational achievement has been associated with a greater neuronal reserve, as well as an increased number of synapses and improved cerebral vascularization, all of which have also been found to contribute significantly to cognitive recovery from several types of acquired brain injury.^16^ Studies have also suggested that higher education levels may lead to better cognitive performance as well as better cognitive and brain reserve,^17,18^ while lower educational attainment may be associated with neuropsychological test patterns similar to those observed in cases of neurological impairment.^19^ Lastly, studies have also found that sociodemographic factors, such as education, may interact with age to provide a protective effect against cognitive aging.^13^

Recently, reading and writing habits have also been found to exert an impact on neurocognitive performance. The quality of what is read and written by participants, their reading proficiency,^20^ as well as the frequency with which individuals engage in reading and writing^9,10^ may all have an influence on cognitive ability. In fact, some authors claim that reading and writing habits may contribute more significantly than the number of years of formal education to neurocognitive performance.^21^ In light of these findings, it has been suggested that the frequency of reading and writing habits (FRWH) may enhance the effects of education on cognition,^9^ especially in individuals with higher educational levels.^10^ However, most studies on the association between education, FRWH and cognition have focused on general intellectual ability rather than specific cognitive components. Furthermore, the literature on cognitive reserve tends to be biased toward the study of dementia, and therefore, focuses predominantly on memory and IQ rather than other cognitive functions.^18^

Given their complexity, relevance to activities of daily living and close interaction with most cognitive components, the executive functions (EF) are among the most extensively studied cognitive functions in the current cognitive and clinical neuropsychological literature. The EF consist of goal-oriented cognitive functions such as problem solving, planning, resistance to distraction, cognitive flexibility, initiation and inhibition.^22^ These are multimodal and multifactorial abilities involved in the processing of both verbal^23,24^ and visuospatial stimuli,^25^ although their role in the latter has been far more widely explored than in the former.

The involvement of EF in verbal processing has traditionally been studied through tasks requiring lexical-semantic processing, such as verbal fluency and the Hayling Test.^26,27^ Performance on the Hayling Test relies heavily on inhibitory control, cognitive flexibility, and verbal processing, both for the generation of context-appropriate responses (in part A of the task) as well as for the suppression of semantically primed words (in part B of the task). This task has been successfully used in the assessment of several neurological populations, and has produced especially interesting results when used in combination with tests involving different input and output modalities, such as the Wisconsin Card Sorting Test. This and other related tasks, such as the Modified Wisconsin Card Sorting Test (MWCST),^28^ rely more heavily on the use of visuospatial planning, behavioral inhibition and rule maintenance.

Card sorting tests have been widely used to assess executive components such as cognitive flexibility, planning and categorization. Successful performance on these tasks requires the involvement of several cognitive processes such as working memory, processing speed and set-shifting.^29,30^ Additionally, like other non-verbal tasks, card sorting tests involve the mental simulation of outcomes. This process is closely related to EF in that it requires the selection of a specific stimulus, the inhibition of surrounding distractors and inappropriate responses, as well as the ability to shift between possible cognitive strategies.^30,31^

Studies of the association between EF and individual and sociocultural variables have found that the inhibition of prepotent responses^30^ is strongly influenced by age.^32^ Age-related inhibition impairment has been found to influence several other cognitive abilities, such as attention, learning^33^ and memory.^34^ Inhibitory control also appears to be impacted by education level.^26^ However, no studies have assessed the influence of the FRWH on inhibitory control using tasks such as the Hayling Test.

In addition to inhibitory control, another key executive component discussed in the literature consists of set-shifting.^30^ This EF is intimately related to cognitive flexibility, and has been described as the ability to alternate between problem-solving strategies or interrupt ineffective behaviors in favor of more adaptive ones.^35^ Like inhibitory control, cognitive flexibility has also been found to be positively correlated with education by several studies investigating the association between sociocultural variables and executive performance.^36^

The occurrence of age-related executive decline has been well documented in the literature;^37^ however, the influence of the FRWH and its association with that of education has only been scarcely studied. One investigation found that older individuals with higher educational attainment and reading levels as well as better vocabulary make more efficient use of their neural resources than those with lower educational and reading levels, and poorer vocabulary.^38^ The authors also found that these variables were associated with performance on measures of EF. A second study has also found that higher reading levels were associated with improved executive performance.^39^ Lastly, a third study assessed the relationship between cognitive functioning and the FWRH and found that a higher frequency of reading and writing combined with higher educational levels were associated with improved executive functioning.^10^ However, it is important to note that each of the aforementioned studies focused on different executive components, such that the findings precluded identification of which specific components are more influenced by the FRWH.

Although a large number of studies have focused on the assessment of EF, few investigations have assessed the impact of sociocultural factors other than education on these cognitive abilities. This is especially true for the FRWH, which has only been scarcely studied in the current literature. Therefore, the goal of the present study was to assess the predictive role of sociocultural variables, such as education and the FRWH, on the cognitive flexibility, initiation, inhibition, processing speed and planning abilities of healthy elderly individuals, as assessed by the MWCST and the Hayling Test.

## METHOD

**Sample.** Fifty-seven healthy adults (n=46; 80.7% females) aged between 60 and 75 years and with 2 to 23 years of formal education were included in the sample. Participants were recruited by convenience from community, workplace and university settings. Only Brazilian-born and native speakers of Brazilian Portuguese, with no uncorrected sensory deficits or any signs of dementia according to the Mini-Mental State Examination,^40,41^ were included in the study. Participants with a current or prior history of alcohol-related problems, illicit drug or benzodiazepine use were excluded from the sample. The sociodemographic data of the sample is given in Table 1.

**Table 1:** Participant sociodemographic data.

Variables	M	SD
Age	66.37	3.90
Education (years)	10.46	5.89
FRWH	13.27	4.74
MMSE	26.54	2.83
SES	6.43	11.59

SD: standard deviation; FRWH: frequency of reading and writing habits; MMSE: Mini-Mental State Examination; SES: socioeconomic status, according to the Brazilian Economic Classification Criteria (42).

**Material and method.** Participants took part in individual assessment sessions lasting approximately 45 minutes each. The present study was approved by the Research Ethics Committee of the institution at which it was conducted, and all participants provided written consent prior to enrollment in the study. The following instruments were used for data collection:

*Sociodemographic and health questionnaire*^43^ - This is a self-report instrument used to collect data on variables such as gender, age, years of formal education, socioeconomic status and FRWH, as well as clinical conditions which may influence performance on cognitive tasks, and would, therefore, imply participant exclusion (e.g. neurological and psychiatric conditions, visual or hearing impairment, alcohol-related problems and use of psychoactive drugs). The questionnaire includes the CAGE scale, which screens for symptoms of alcohol dependence (version adapted by Amaral & Malbergier,^44^ a reading and writing inventory, which inquires as to the frequency with which individuals read newspapers, magazines, books or other types of reading material, and write essays, notes or other types of text. Each activity is assigned a score from 0 to 4, where higher scores are indicative of higher frequencies. Item scores are summed for a total ranging from 0 to 28.

*Modified Wisconsin Card Sorting Task (MWCST)*^28^ - The task involves a deck of 48 cards on which geometric figures varying in number, shape and color are printed. Participants must match the cards according to a particular set of rules, which they must identify based on accuracy feedback following each response. The MWCST assesses planning, abstract reasoning, learning, rule maintenance, and cognitive flexibility. In the present study, the total number of categories completed, the number of perseverative and non-perseverative errors, as well as failures to maintain set were analyzed.

*Hayling Test*^27^ - Adapted to Brazilian Portuguese by Fonseca, Oliveira, Gindri, Zimmermann & Reppold.^45^ This is a two-part assessment instrument, consisting of two sets of 15 sentences in which the last word is omitted. The participant is asked to complete each sentence as quickly as possible with a word that logically fits the sentence (Part A) or with a word which is semantically unrelated to the sentence (Part B). Cognitive functions assessed by this instrument include processing speed, initiation, inhibitory control and cognitive flexibility. In the present study, the latency of responses to the stimuli in Parts A and B of the test, the number of correct and incorrect responses, as well as the difference between the time taken to complete each part of the test were analyzed.

**Data analysis.** Data were analyzed using the SPSS software package, version 17.0. Kolmogorov-Smirnov tests were used to identify deviations from normal distributions, and Pearson and Spearman coefficients were used to assess the relationship between parametric and nonparametric variables, respectively. These results were used to construct a multiple linear regression model with neuropsychological performance as the dependent variable and education and FRWH as independent factors. Results were considered significant at 5%.

## RESULTS

Participant scores on the Hayling Test and MWCST are given in Table 2.

**Table 2:** Participant scores on the Hayling Test and MWCST.

Variable	M	SD
MWCST Categories Completed(/6)	4.14	1.89
MWCST Perseverative errors	12.30	10.05
MWCST Non-perseverative errors	3.37	2.60
MWCST Failures to maintain set	0.56	0.80
Hayling Test Part A speed	19.12	6.48
Hayling Test Part A errors (/15)	0.54	0.93
Hayling Test Part B speed	61.16	29.61
Hayling Test Part B errors (/15)	6.96	3.47
Hayling Test Part B speed - Part A speed	41.90	26.59
Hayling Test Part B speed/ Part A speed	3.28	1.33

The results of correlations between performance on the two tasks and participants' education and FRWH are shown in Table 3.

**Table 3:** Correlation coefficients between education, FRWR, and performance on the Hayling Test and MWCST.

	Education (Years)	FRWH
MWCST - Categories	0.544**	0.438**
MWCST - P Errors	-0.507**	-0.381**
MWCST - NP Errors	-0.347**	-0.291*
MWCST - FMS	-0.258	-0.271*
Hayling A - Speed	-0.398**	-0.213
Hayling A - Errors	-0.112	-0.056
Hayling B - Speed	-0.316*	-0.121
Hayling B - Errors	-0.377**	-0.187
Hayling Speed B-A	-0.293*	-0.053
Hayling Speed B/A	-0.074	0.133

FRWH: Frequency of reading and writing habits; MWCST: Modified Wisconsin Card Sorting Task; MWCST-FMS: Modified Wisconsin Card Sorting Task - Failure to Maintain Set.

* p<0.05;

** p<0.01.

The results in Table 3 suggest that both the number of years of formal schooling completed by each participant as well as their FRWH were weakly to moderately correlated with the number of categories completed on the MWCST, and inversely associated with the number of perseverative and non-perseverative errors on the task. Interestingly, only the FRWH was significantly correlated with the number of failures to maintain sets. Speed and accuracy on the Hayling Test were only correlated with participant educational levels. Based on these results, a multiple linear regression model was constructed to assess the effects of education and the FRWH on scores in both of these tasks. The results of this analysis are shown in Table 4.

**Table 4:** Multiple linear regression model of performance on the Hayling Test and MWCST according to education and FRWH of healthy elderly subjects.

Variables		B	SE (B)	b	R^2 ^
MWCST Categories	Education	0.129	0.041	0.396	0.289**
	FRWH	0.094	0.051	0.236	
MWCST P Errors	Education	-0.555	0.235	-0.318	0.198*
	FRWH	-0.441	0.286	-0.208	
MWCST NP Errors	Education	-0.108	0.063	-0.239	0.137*
	FRWH	-0.110	0.077	-0.201	
MWCST FMS	FRWH	-0.030	0.023	-0.177	0.031
Hayling A Speed	Education	-0.421	0.137	-0.383	0.147*
Hayling B Speed	Education	-1.651	0.639	-0.331	0.110*
Hayling B Errors	Education	-0.203	0.075	-0.345	0.119*
Hayling Speed B-A	Education	-1.232	0.585	-0.275	0.076*

FRWH: Frequency of reading and writing habits; MWCST: Modified Wisconsin Card Sorting Task; MWCST-FMS: Modified Wisconsin Card Sorting Task - Failure to Maintain Set.

* p<0.05;

** p<0.01.

Education and FRWH accounted for 28.9% of the variability in the number of completed categories in the MWCST, 19.8% of the number of perseverative errors and 13.7% of the non-perseverative errors on the task. Interestingly, the combination of these two variables had a greater predictive impact on performance on this task than either of the two variables alone (data not shown). On the other hand, the variability in scores on the Hayling Test was best accounted for by participant education, which accounted for 14.7% of the speed and 11% of the accuracy in part A of the task, and 11.9% of the accuracy in part B of the instrument, as well as 7.6% of the discrepancy between the time taken to complete both parts of the test.

## DISCUSSION

The aim of the present study was to assess the predictive impact of the sociocultural factors education and FRWH on the EF of healthy elderly. Cognitive flexibility and planning were assessed by the MWCST, a predominantly visuospatial task, while initiation, inhibition, processing speed and cognitive flexibility were evaluated using the Hayling Test, a two-part verbal assessment instrument. Although scores on both tasks appeared to be influenced by the number of years of formal education and FRWH, the combination of these two variables appeared to have a more significant impact on the EF underlying performance on the MWCST.

These findings suggested that, while education had a more significant influence on verbal initiation, inhibition and cognitive flexibility, both education and the FRWH were found to influence planning, working memory, short-term memory and cognitive flexibility, as assessed by a non-verbal instrument. As such, our hypothesis that the two sociocultural variables influence performance on both tasks, but have a greater impact on Hayling Test scores due to the verbal nature of its stimuli, was only partially confirmed. Although both variables had some influence on performance on the Hayling Test, only education was able to accurately predict the variability in its scores. The relative linguistic simplicity of the Hayling Test may have accounted for these results, since sentence completion may be considered an automatic language task for elderly individuals. This hypothesis is supported by studies which show that, although some language abilities may decline with age,^46^ several cognitive-communicative processes remain stable over time.^47^ However, we believe that if a discourse processing task had been used as an assessment tool for verbal EF, its increased complexity and the associated increase in cognitive demand (due to the need to access verbal language beyond the word level, and verbal working memory overload) may have suffered a greater influence from the FRWH.^48^

On the other hand, on the MWCST, the two variables were found to have a greater predictive impact when analyzed in combination rather than individually. Previous studies involving the same neuropsychological instruments have found that both age and education influenced performance on the tasks, with elderly individuals performing worse than younger participants, and subjects with higher education levels obtaining superior scores than those with lower educational attainment.^32,49^ Other studies have also found correlations between performance on highly complex discourse tasks with significant executive demands and scores on the WCST, suggesting that, even though the latter is a non-verbal task, it relies on the same cognitive abilities underlying verbal tests.^23^

However, no studies have assessed the combined impact of education and the FRWH on performance in these tasks among elderly individuals. Furthermore, the few studies which have looked into the beneficial influence of reading habits on EF did not use the same assessment tasks as the present study, and assessed reading level based on proficiency tests rather than on the reported frequency of reading and writing.^38,39^ The only study which has assessed reading and writing habits in the same way as the present investigation used different instruments to assess EF, namely, verbal fluency and simple problem-solving tasks.^10^ These limitations preclude the comparison of the present findings with those of other investigations in the literature.

The FRWH is an ecological measure of cognitive stimulation and allows for an assessment of current intellectual activity, whereas education levels only indicate the duration of previous periods of continuous cognitive stimulation, which may have occurred long before participants are assessed as part of a neuropsychological study. Although the FRWH may not have an especially relevant impact on less complex verbal tasks, it may predict performance on more complex or non-verbal tasks, as shown by the present findings. The Hayling Test, which predominantly involves sentence completion, is simpler for both discourse and verbal fluency tasks, both of which rely more heavily on executive processes, and would therefore be more likely to be influenced by the FRWH.

In conclusion, it appears that, in this sample of elderly subjects, cognitive flexibility was sufficiently preserved to allow for adequate performance on verbal tasks, mainly due to their decreased reliance on complex verbal executive processing. However, for the successful completion of complex non-verbal tasks, elderly participants may have benefitted from the additional stimulation provided by regular reading and writing habits. Although education still seems to be the most relevant sociocultural factor for EF in old age, the present findings suggest that the FRWH must be taken into account in combination with the education variable when more complex cognitive functions are considered.

The present study provided preliminary evidence of the impact of sociocultural factors on EF in aging, highlighting the role of education in simpler tasks and the combined influence of this variable and FRWH in more complex ones. The present findings must be interpreted in light of some limitations, such as the inherent difficulties in assessing participant education, and the problems associated with measuring the frequency and quality of reading and writing habits. However, in spite of these limitations, our findings showed that the importance of the quality of elderly individuals' educational background and of continuous cognitive stimulation through reading and writing after exposure to formal education must be further explored. Additionally, the interaction between these factors in both healthy elderly and clinical populations should be investigated in future studies.
